# 2072. Clinical Evaluation of the BioFire® Global Fever Special Pathogens Panel

**DOI:** 10.1093/ofid/ofad500.142

**Published:** 2023-11-27

**Authors:** Brittany Collins, David S Rabiger, Mark Gurling, Jared R Helm, Wendy Smith, Pascal Belgique, Olivia Jackson, Michael Johnson, Alex J Kelley, Hannah VanHollebeke, Sidney Webster, Ashley Wiltsie, Marianne Kim, Cynthia L Phillips

**Affiliations:** BioFire Defense, LLC, Salt Lake City, UT; BioFire Defense, Salt Lake City, Utah; BioFire Defense, LLC, Salt Lake City, UT; BioFire Defense LLC, Riverton, Utah; BioFire Defense, LLC, Salt Lake City, UT; BioFire Defense, Salt Lake City, Utah; BioFire Defense, Salt Lake City, Utah; BioFire Defense, LLC, Salt Lake City, UT; BioFire Defense, LLC, Salt Lake City, UT; BioFire Defense, LLC, Salt Lake City, UT; BioFire Defense, LLC, Salt Lake City, UT; BioFire Defense, LLC, Salt Lake City, UT; BioFire Defense, LLC, Salt Lake City, UT; BioFire Defense, Salt Lake City, Utah

## Abstract

**Background:**

BioFire Defense has developed the BioFire® Global Fever Special Pathogens Panel, in collaboration with the U.S. Department of Defense and NIAID (contract Nos. W911QY-13-D-0080 and HHSN272201600002C), as an in vitro diagnostic test for the simultaneous identification of viral, bacterial, and protozoan pathogens directly from whole blood in under an hour using the BioFire® FilmArray® platform. The BioFire Global Fever Special Pathogens Panel has been cleared by the U.S. FDA for the detection of chikungunya virus, Crimean-Congo hemorrhagic fever virus (CCHFV), dengue virus, (serotypes 1-4), *Ebolavirus* spp., Lassa virus, Marburg virus, West Nile virus (WNV), yellow fever virus (YFV), *Bacillus anthracis*, *Francisella tularensis*, *Leptospira* spp., *Yersinia pestis, Leishmania* spp., and *Plasmodium* spp. from individuals with signs and/or symptoms of acute febrile illness (AFI) and known or suspected exposure to target pathogens.

**Methods:**

BioFire Defense performed a prospective clinical evaluation for the Global Fever Special Pathogens Panel at eleven geographically distinct sites across the globe. A total of 2139 prospective whole blood specimens were tested between March 2018 and March 2021.

**Results:**

The Global Fever Special Pathogens Panel detected at least one analyte in 33.9% of specimens (725/2139). The most prevalent analyte was *Plasmodium* (29.0%, 118/407), followed by dengue virus (36.4%, 266/730). Chikungunya virus, *Leptospira* spp., *Leishmania* spp., CCHFV, and WNV were detected in fewer than 5% of specimens. Two or more analytes were detected in 4.3% (31/725) of positive specimens. All other analytes were not detected by the Global Fever Special Pathogens Panel. Positive percent agreement between the Global Fever Special Pathogens Panel and comparator testing ranged between 92.7-100%, and the negative percent agreement ranged between 99.2-100%.

**Conclusion:**

The results show that the BioFire Global Fever Special Pathogens Panel could aid in rapid and actionable AFI diagnosis caused by multiple, sometimes co-occurring, pathogens. The Global Fever Special Pathogens Panel will be a valuable diagnostic tool to aid in national preparedness for disease outbreaks.

**Disclosures:**

**All Authors**: No reported disclosures

